# Supplementary figures and images for: Genome-wide association study of leprosy in Malawi and Mali
Source: PLoS Pathog. 2022 Sep 19;18(9):e1010312. doi: 10.1371/journal.ppat.1010312 (PMC9624411; doi:10.1371/journal.ppat.1010312)

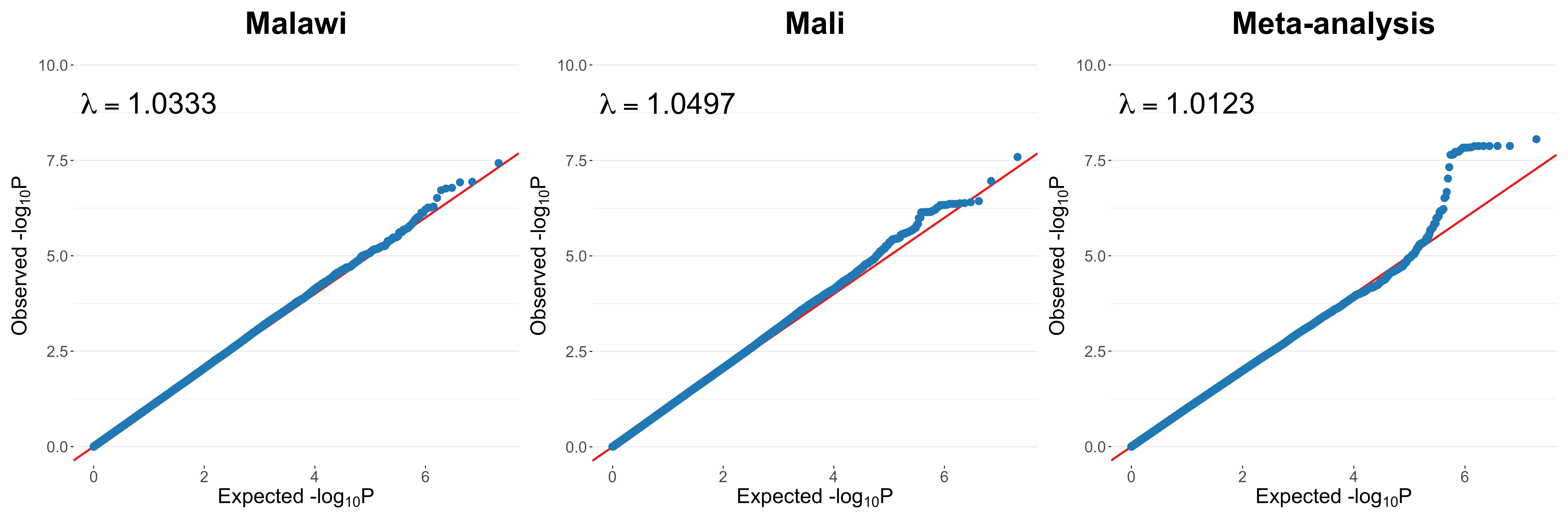

Supplement: S1 Fig — QQ-plots of leprosy association in Malawi (284 cases, 328 controls, SNPs = 10,511,695), Mali (208 cases, 311 controls, SNPs = 10,514,676) and fixed-effects meta-analysis of both populations (cases = 492, controls = 639, SNPs = 9,616,523. (TIF) [file ppat.1010312.s001.tif]

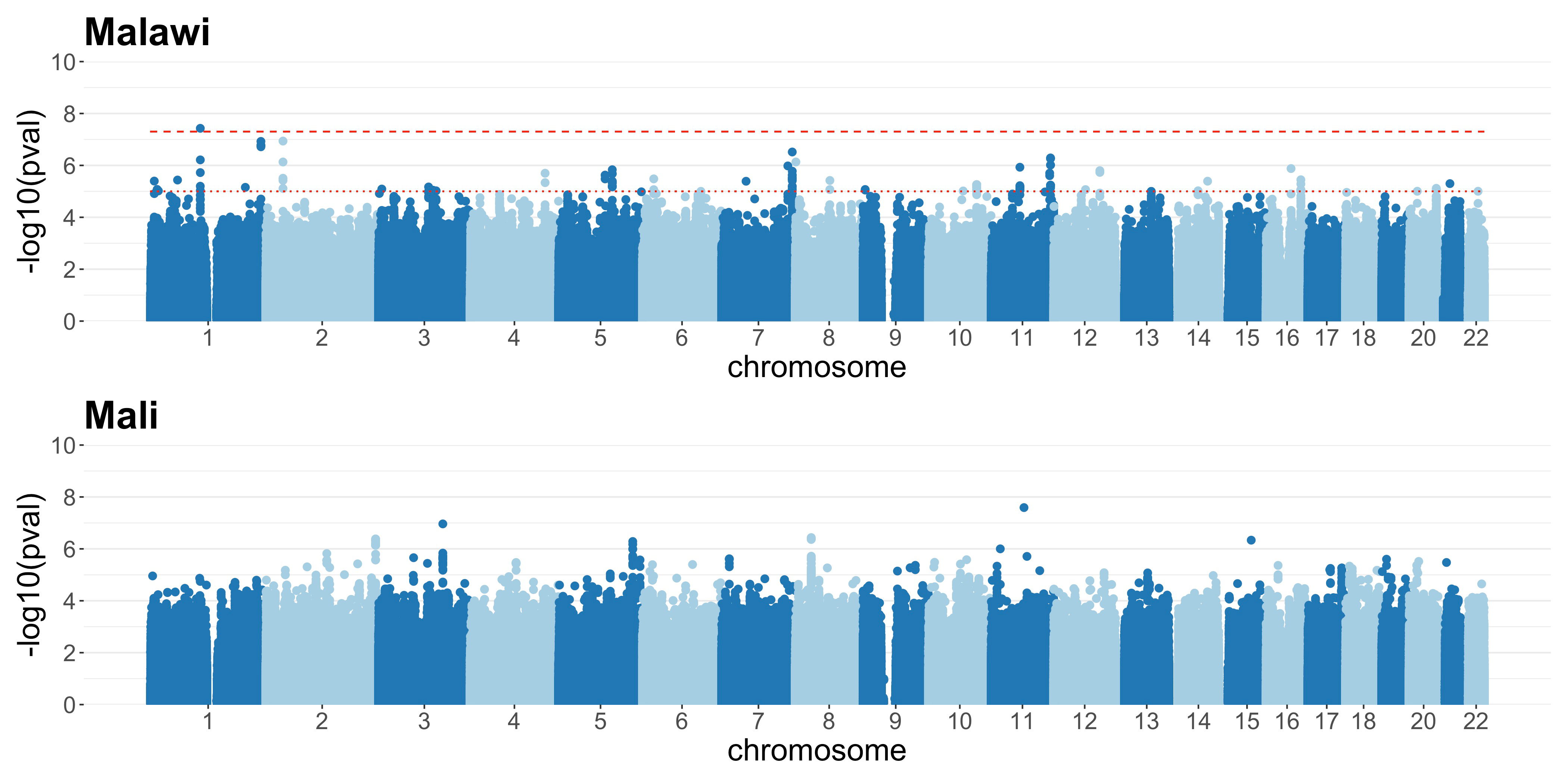

Supplement: S2 Fig — Manhattan plots of leprosy association in discovery (Malawi, 284 cases, 328 controls, SNPs = 10,511,695) and replication (Mali, 208 cases, 311 controls, SNPs = 10,514,676) samples. P-value thresholds are annotated on the Malawi Manhattan plot: dashed line, p = 5 × 10^−8^ (genome-wide significance); dotted line, p = 1 × 10^−5^ (threshold for suggestive association). In Malawi, 142 SNPs exceed a significance threshold p = 1 × 10^−5^ and 1 exceeds p = 5 × 10^−8^. In Mali 176 SNPs exceed a significance threshold p = 1 × 10^−5^ and 1 exceeds p = 5 × 10^−8^. (TIF) [file ppat.1010312.s002.tif]

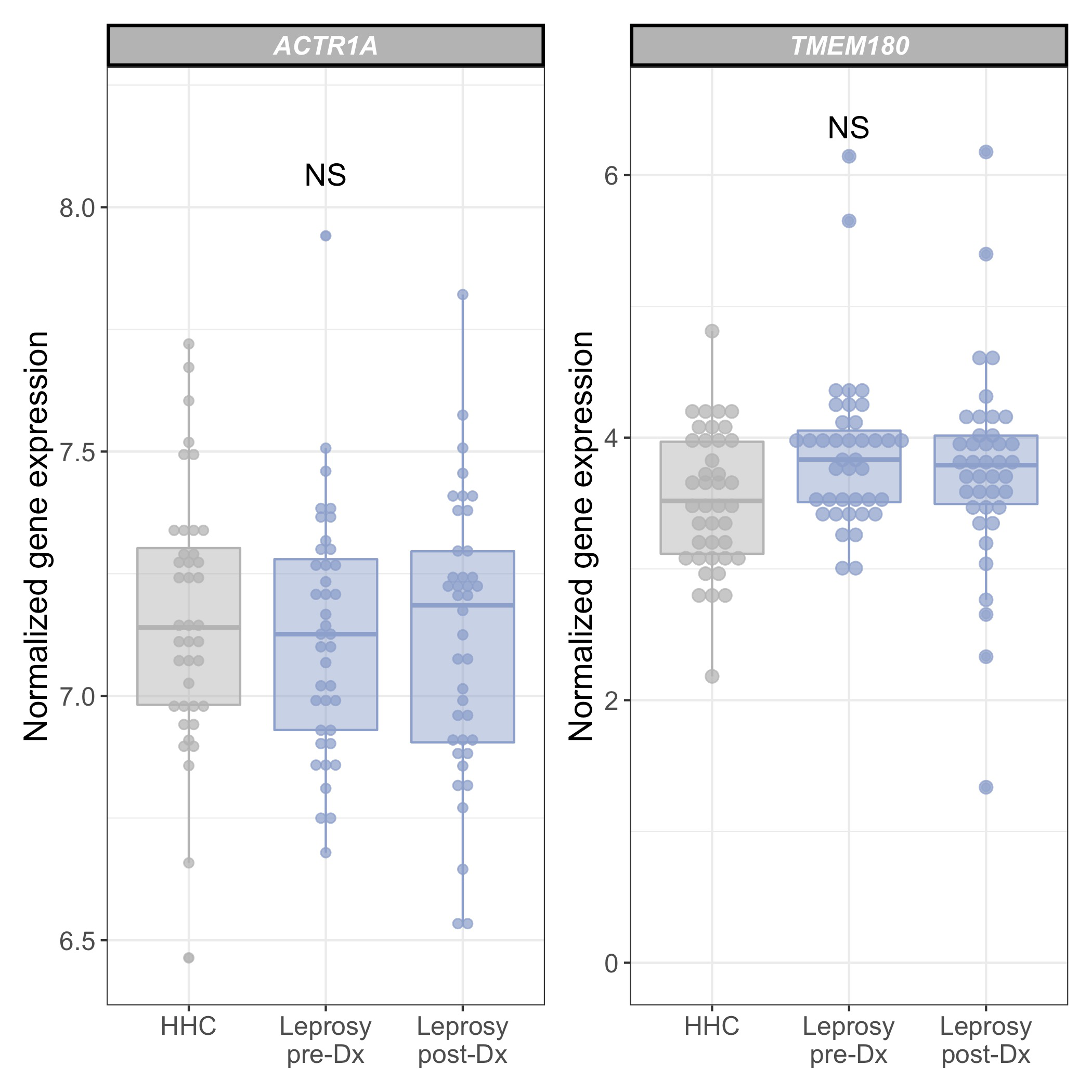

Supplement: S3 Fig — ACTR1A and TMEM180 expression in whole blood of household contacts who do not develop leprosy (HHC, n = 40) and those that do (n = 40); before diagnosis (Leprosy pre-Dx) and at the point of diagnosis (Leprosy post-Dx). Data is taken from the Tio-Coma et al RNA-Seq dataset [25] (GEO, GSE163498). Gene expression between groups is compared by ANOVA (ACTR1A), or Kruskal-Wallis tests (TMEM180) if the data was non-normally distributed. (TIF) [file ppat.1010312.s003.tif]

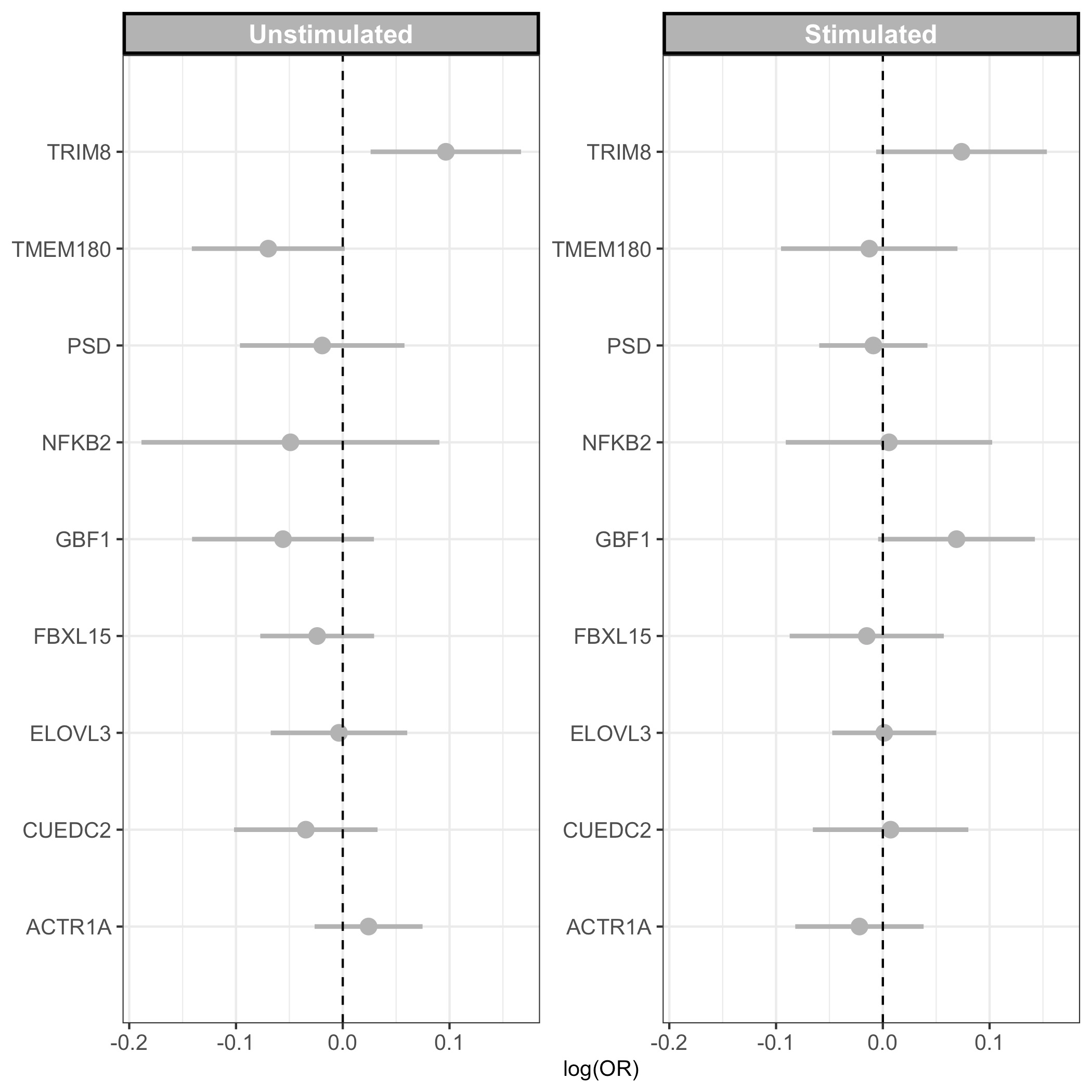

Supplement: S4 Fig — Effect of the rs2015583:G allele on gene expression at 9 genes in cis (within a 500kb window) to rs2015583 in whole blood from leprosy patients (n = 51) with (left) and without (right) stimulation with sonicated M. leprae. Data is taken from the Manry et al dataset [24] (GEO, GSE100853). Genotype at rs2015583 is correlated with gene expression using linear regression, correcting for 7 principal components of gene expression data in the unstimulated samples and 8 in the stimulated samples. Gene expression is not significantly associated (p < 0.05) with rs2015583 genotype for any gene with or without stimulation. (TIF) [file ppat.1010312.s004.tif]

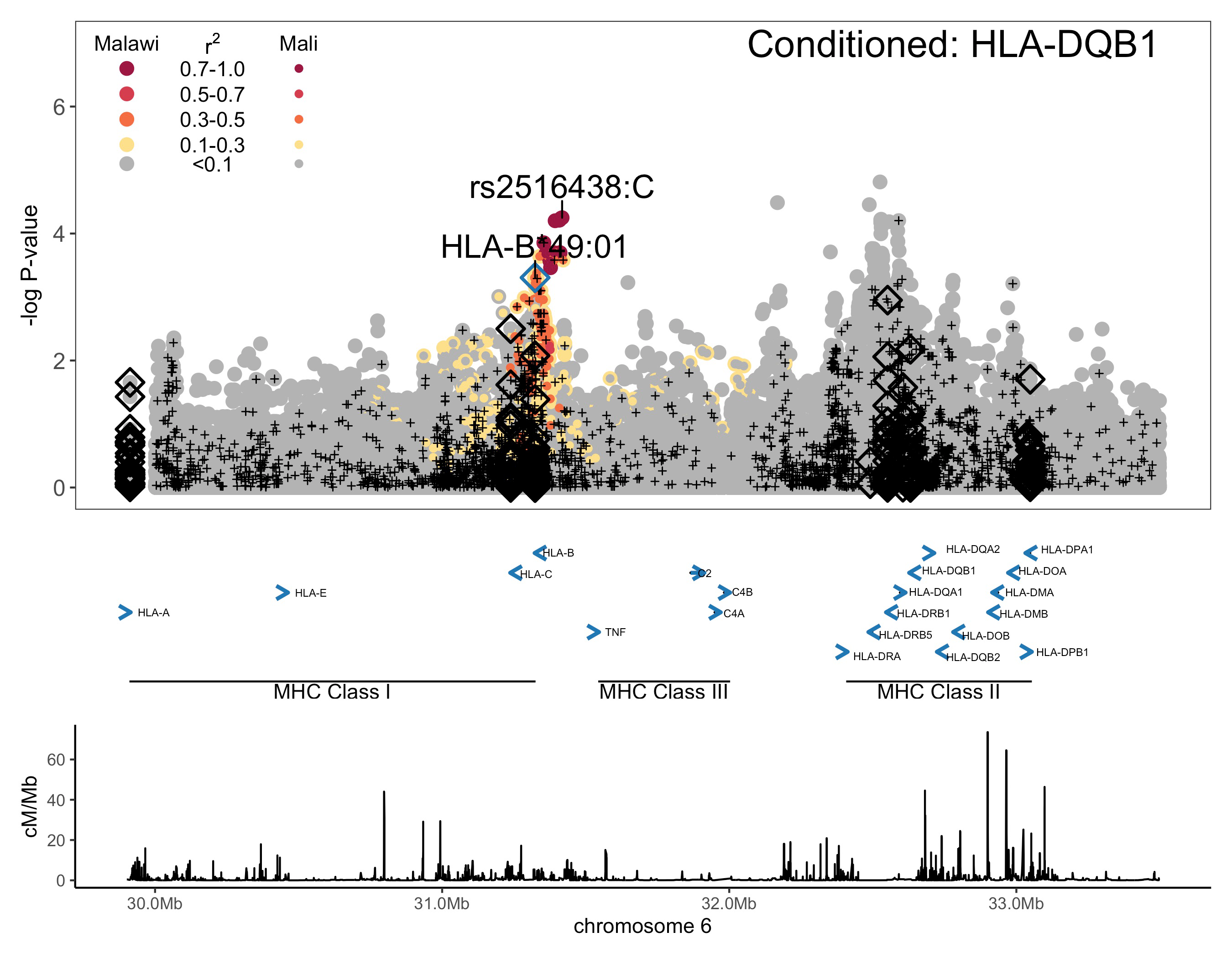

Supplement: S5 Fig — Association statistics represent a fixed-effects meta-analysis of additive association with disease in Malawi and Mali conditioned on HLA-DQB1*04:02. SNPs are coloured according to linkage disequilibrium to rs2516438, and genotyped SNPs marked with black plusses. Imputed classical HLA alleles are plotted as diamonds, with significantly associated (FDR <0.05) alleles highlighted in blue. (TIF) [file ppat.1010312.s005.tif]

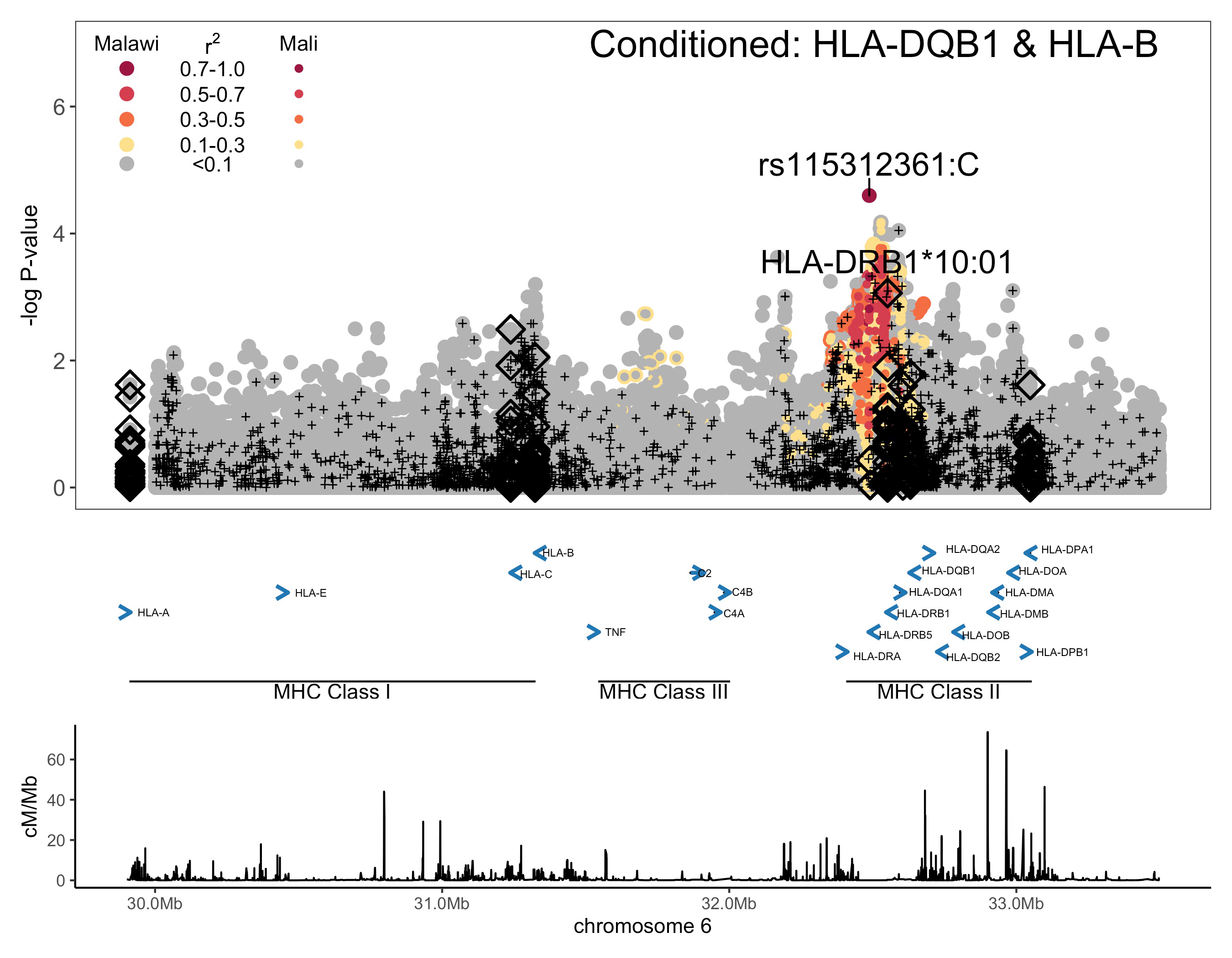

Supplement: S6 Fig — Association statistics represent a fixed-effects meta-analysis of additive association with disease in Malawi and Mali conditioned on HLA-DQB1*04:02 and HLA-B*49:01. SNPs are coloured according to linkage disequilibrium to rs115312361, and genotyped SNPs marked with black plusses. Imputed classical HLA alleles are plotted as diamonds. No significantly associated (FDR <0.05) alleles remain after conditioning on HLA-DQB1*04:02 and HLA-B*49:01. (TIF) [file ppat.1010312.s006.tif]

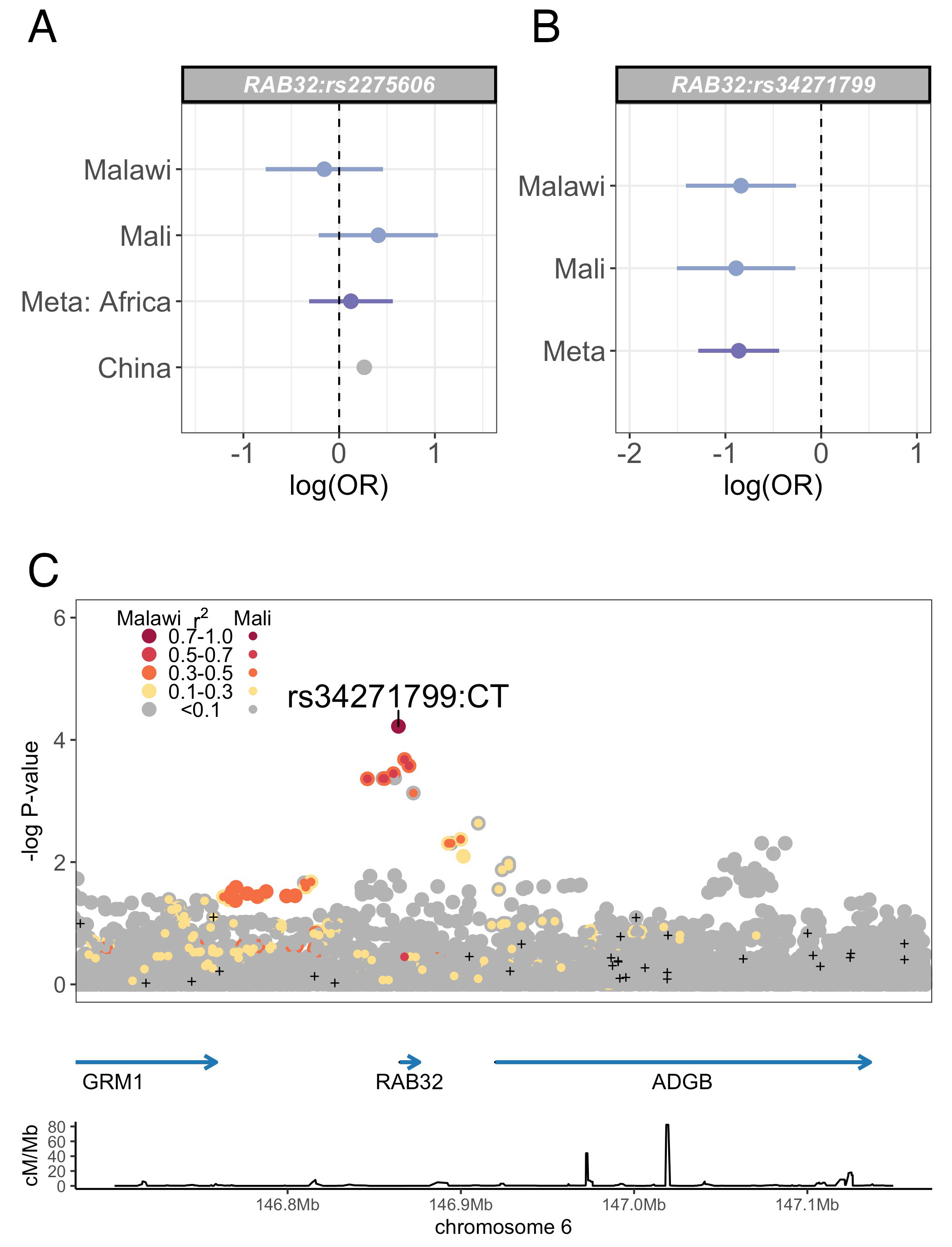

Supplement: S7 Fig — (A) Log-transformed odds ratios and 95% confidence intervals of rs2275606 association (peak association in Chinese GWAS data) with leprosy in Malawi, Mali and China. (B) Log-transformed odds ratios and 95% confidence intervals of rs34271799 association with leprosy in Malawi and Mali. (C) Regional association plot of leprosy association at the RAB32 locus. Association statistics represent a fixed-effects meta-analysis of additive association with disease in Malawi and Mali. SNPs are coloured according to linkage disequilibrium to rs34271799, and genotyped SNPs marked with black plusses. (TIF) [file ppat.1010312.s007.tif]

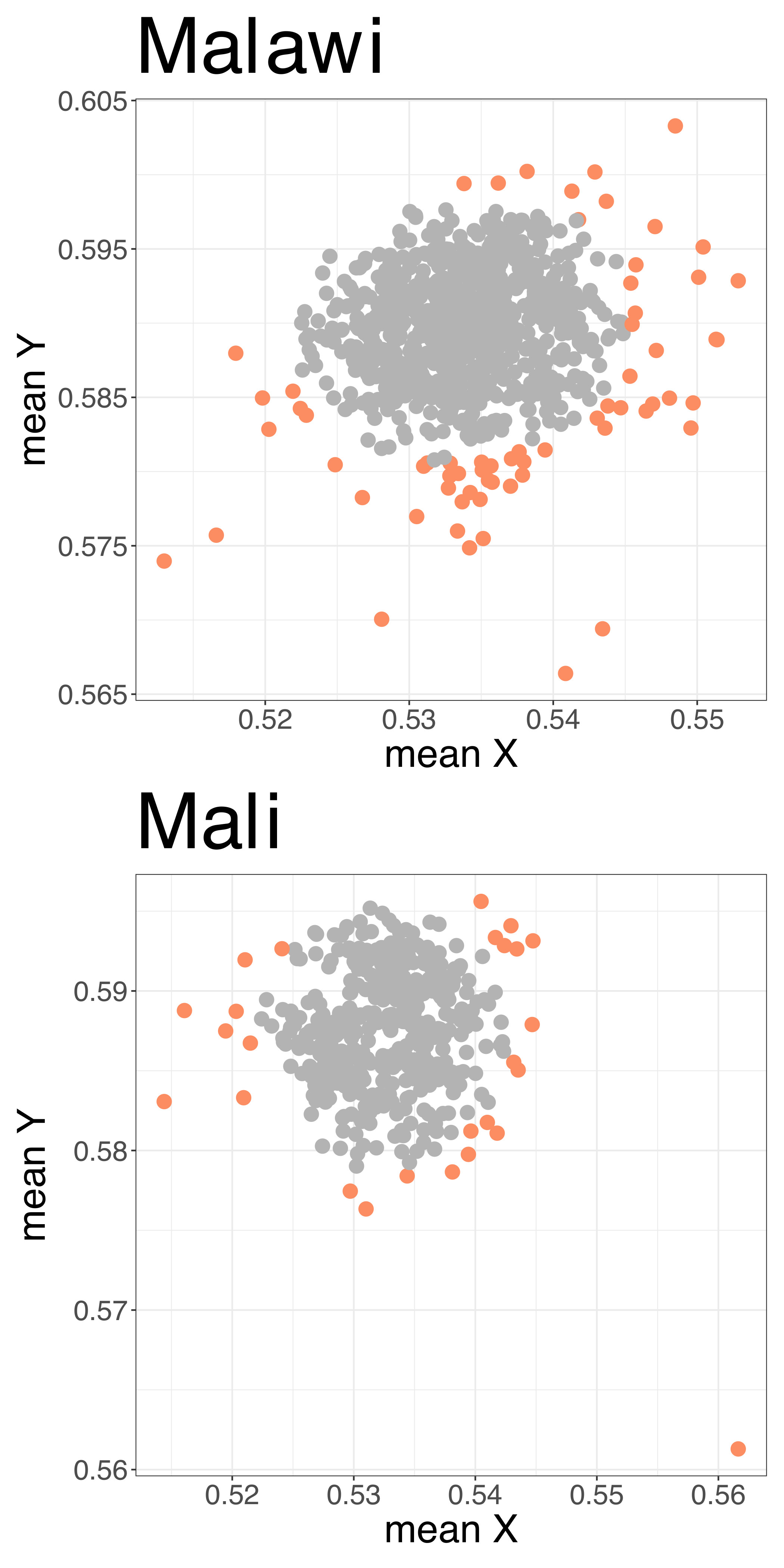

Supplement: S8 Fig — (A) Mean X and Y channel intensities for Malawi (top) and Mali (bottom) samples. Outlying samples were identified using ABERRANT and are highlighted (orange). (TIF) [file ppat.1010312.s008.tif]

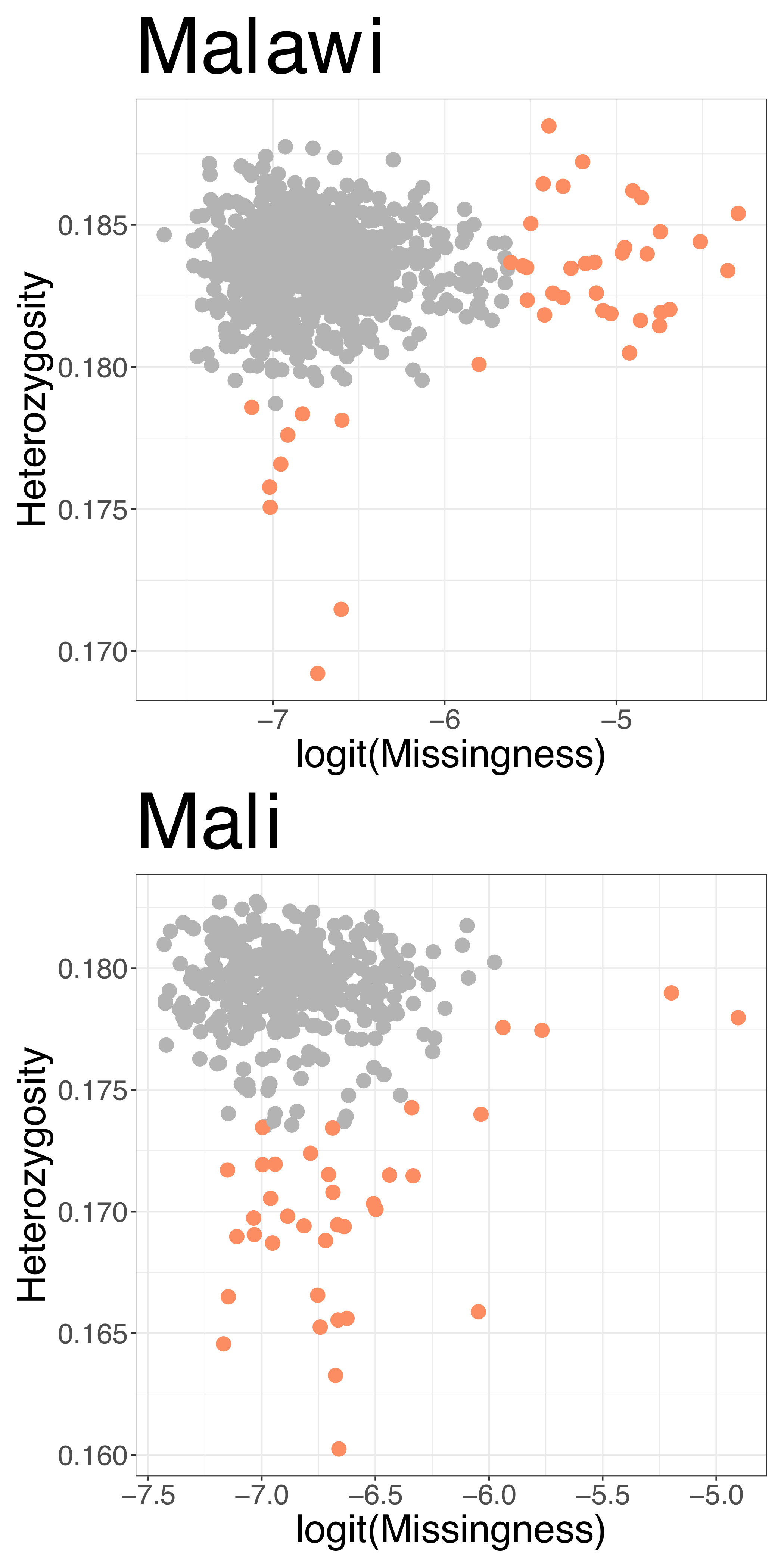

Supplement: S9 Fig — (A) Mean sample genotype missingness plotted against heterozygosity for Malawi (top) and Mali (bottom) samples. Outlying samples were identified using ABERRANT and are highlighted (orange). (TIF) [file ppat.1010312.s009.tif]

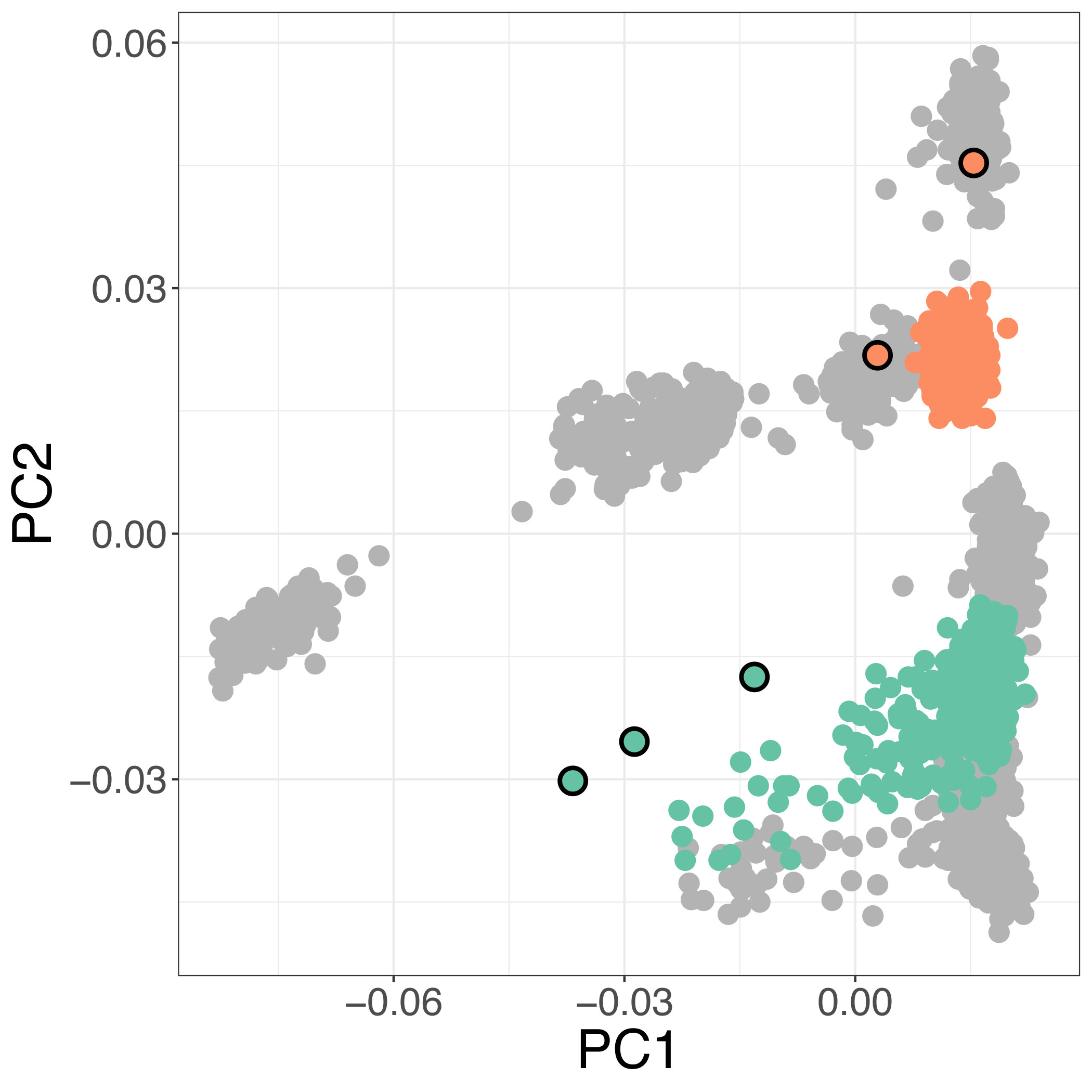

Supplement: S10 Fig — Plot of the major two principal components of genome wide genotyping data. Malawi study samples are plotted in orange and Mali study samples in green, against a background of African Genome Variation Project samples (gray). Outliers are highlighted (black rings). (TIF) [file ppat.1010312.s010.tif]

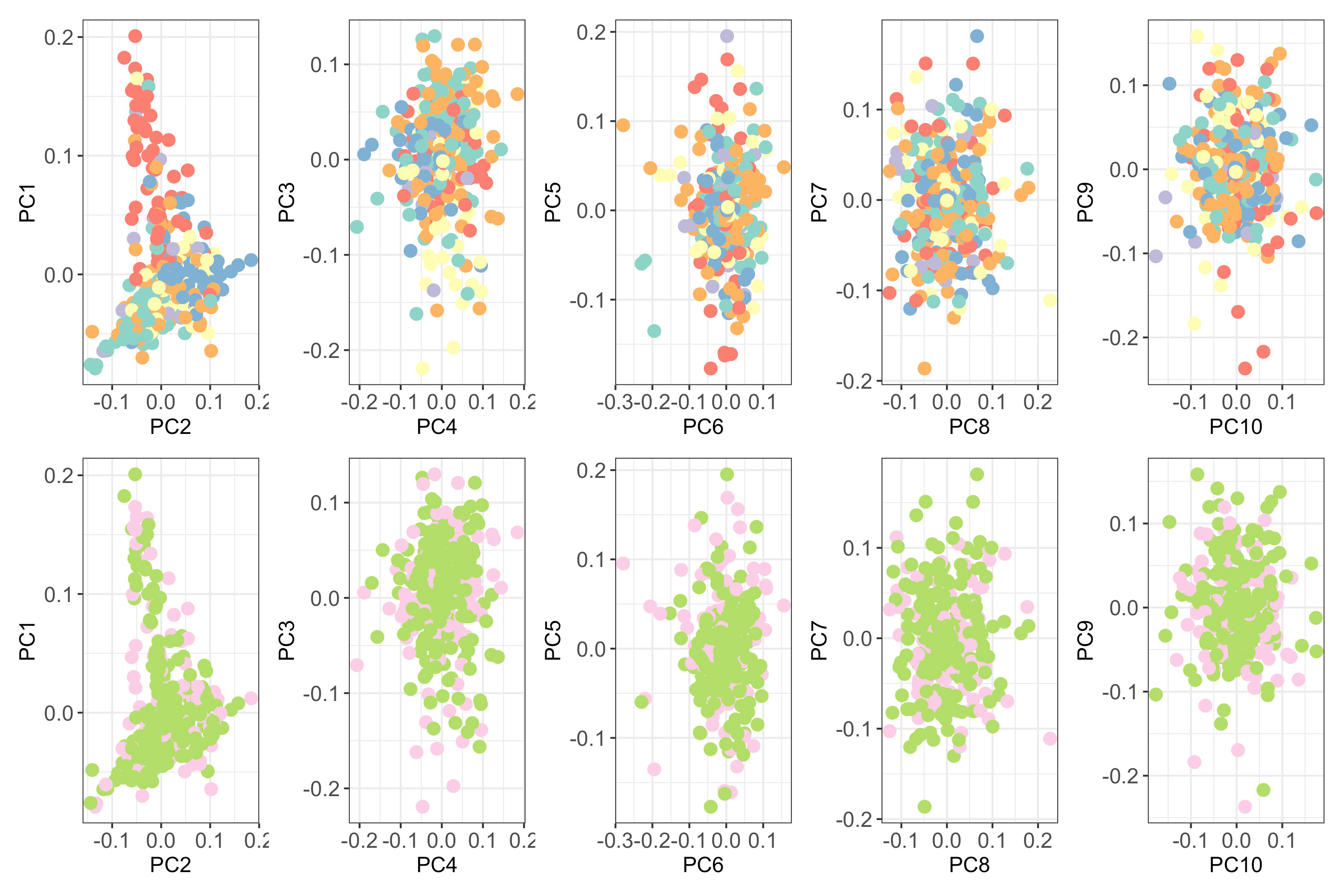

Supplement: S11 Fig — Individuals are color-coded according to self-reported ethnicity (top) and case-control status; cases in pink, controls in green (bottom). (TIF) [file ppat.1010312.s011.tif]

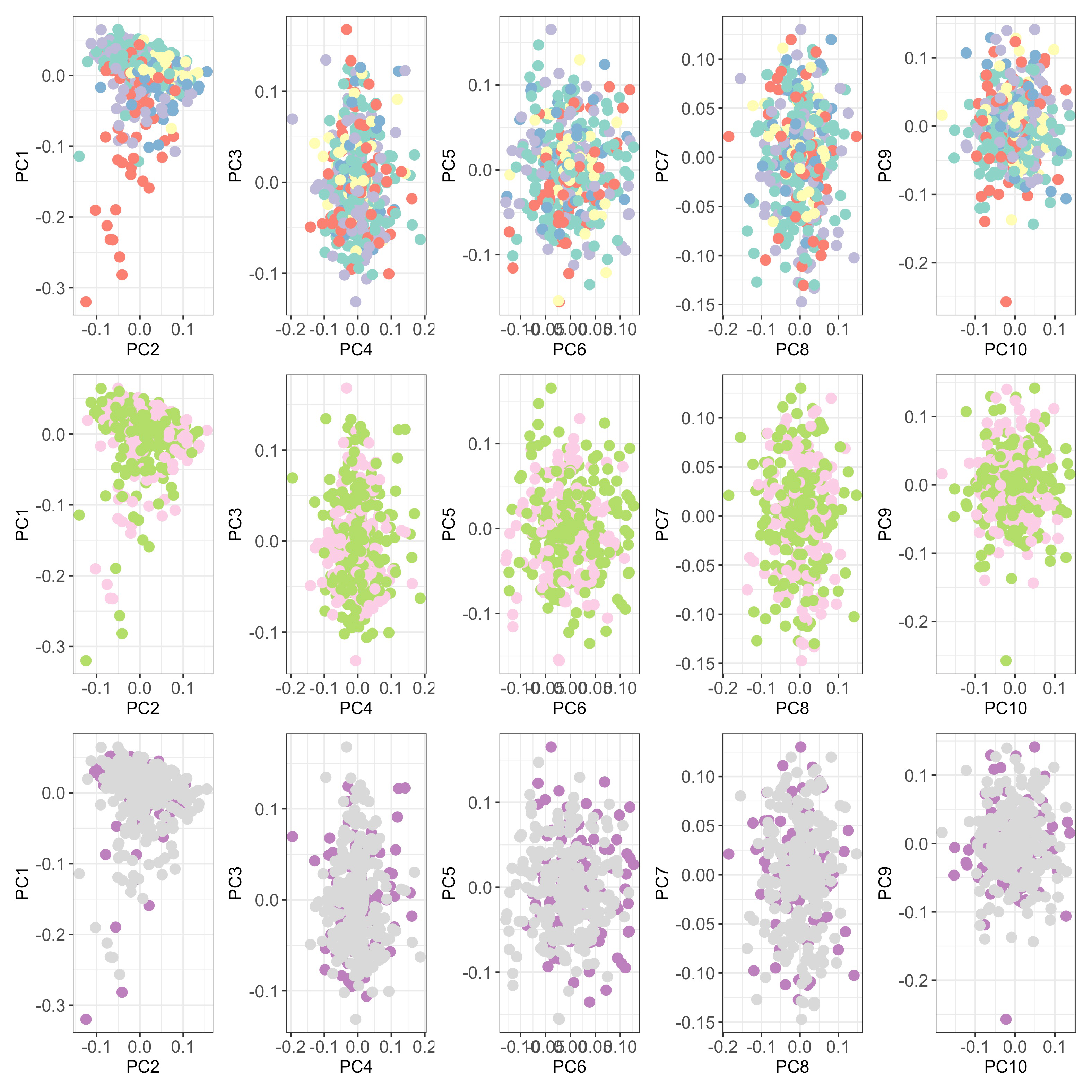

Supplement: S12 Fig — Individuals are color-coded according to self-reported ethnicity (top), case-control status (middle; cases in pink, controls in green), and genotyping platform (bottom; Omni 2.5M in purple, Africa Diaspora Power Chip in gray). (TIF) [file ppat.1010312.s012.tif]

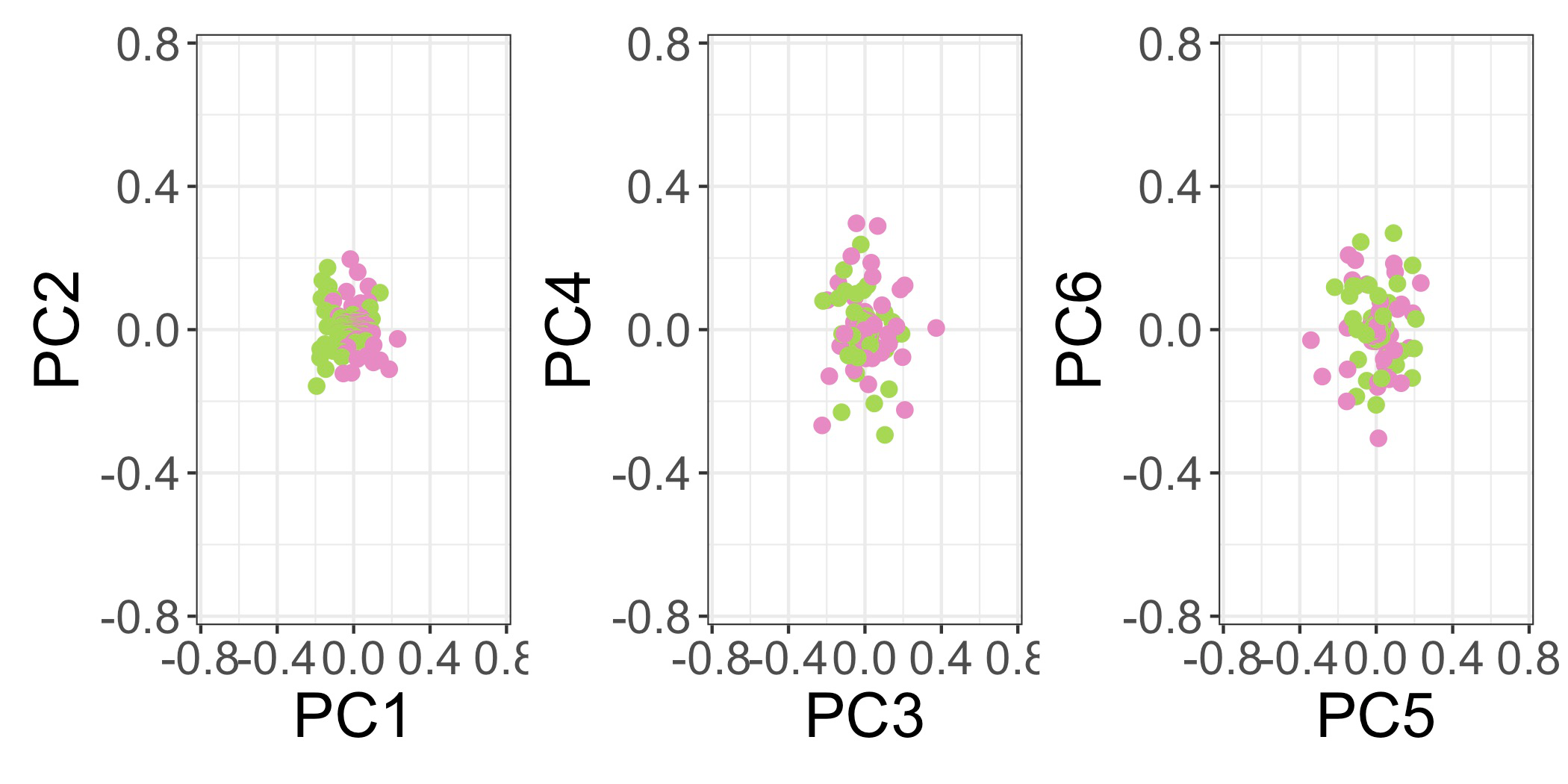

Supplement: S13 Fig — Individuals are color-coded according to case-control status (middle; cases in pink, controls in green). (TIF) [file ppat.1010312.s013.tif]
