# Supplementary material for: Genome-wide association study of leprosy in Malawi and Mali
Source: PLoS Pathog. 2022 Sep 19;18(9):e1010312. doi: 10.1371/journal.ppat.1010312 (PMC9624411; doi:10.1371/journal.ppat.1010312)
Supplement: Attachment — Submitted filename: leprosy_GWAS_response_to_reviewers.docx [file ppat.1010312.s027.docx]

Point-by-point Reviewer Responses

Responses are highlight in bold/italics. Line numbers refer to the manuscript with highlighted changes.

Reviewer #1

Summary

This paper took a look at leprosy susceptibility in African populations by conducting a genome-wide association study, while only one novel leprosy locus was identified and replicated known locus on HLA, LACC1 and SLC29A3 with a nominal significance. This approach is unique and commendable. Generally, the paper is well written, although there are some important points that are not clear that must be addressed.

***Many thanks for taking the time to review our work.***

Major Issues

1. Better to provide a map which described the sample distribution from African population.

***Many thanks for this suggestion. We have included this as panel A to Figure 1.***

2. Sample information summary and statistics of main results should be listed as tables in the main text.

***We now provide sample demographics and clinical characteristics in Table 1 and statistics for the lead variant in Table 2 in the main manuscript.***

3. How to explain that most published GWAS results were not replicated in African leprosy patients? If there was sample size limitation, should perform a power calculation analysis to identify the power to discover novel locus or replicate known locus. If there was population heterogeneity issue, should also take a look at the allele frequency difference between populations.

***Very many thanks for raising this important point. We now expand our discussion and analysis of the contribution of study power to our lack of replication at previously reported leprosy risk loci, extending Table S10 to include an analysis of allele frequency differences between our study populations and the discovery populations for each locus. We have added the following to the text (lines 212-219 and 226-236):***

***“Of 37 leprosy risk loci assessed, 3 were not reliably imputed in our dataset (rs145562243, rs6871626, rs1873613) and 10 (rs146466242, rs925368, rs671, rs149308743, rs142179458, rs75680863, rs2066844, rs76418789, rs5743291, rs663743) were monomorphic or at very low frequency (MAF<1%) in our study samples (Supplementary Table 6). We were thus able to assess replication at 24 loci, at which our study had adequate power (80%) to replicate (p<0.05) findings at 7 loci.”***

***“Our failure to replicate evidence for leprosy association at more than 2 loci reflects a lack of study power at 17 of 24 SNPs assessed (Supplementary Table 6). Our study power is in part a reflection of our modest sample numbers, but is also contributed to by allele frequency differences between discovery populations and our study samples. At 20 of 37 loci, the allele frequency difference between that reported in the discovery analysis and our study samples was >10%, of which 14 resulted in decreased power to detect a genetic association in African populations (Supplementary Table 6). A striking example of this is at the IL18RAP/IL18R1 locus (rs2058660), at which the discovery population MAF is 0.49, falling to 0.10 and 0.11 in Malawi and Mali respectively.”***

4. Current evidence about potential function for the novel locus is only based on published eQTL mapping studies and not enough for the conclusions. The author should perform experiment to validate the eQTL results.

***Very many thanks for raising this point. We have considerably extended our analysis of the potential function of the novel locus, integrating chromatin state data, transcriptomic data from leprosy patients and additional eQTL data from other tissues. See Results sections; “Regulatory function at the leprosy risk locus” (lines 114-147), “Candidate gene expression in leprosy” (lines 149-179), and Methods sections; “Functional annotation and pleitropy” (lines 595-641), “Differential gene expression analysis in leprosy” (lines 643-678).***

5.The title for this manuscript is over estimated. More work on either genetics or biology for IBD and atopy should be performed before concluded that ACTR1A has pleiotropic effects.

***We agree that the original title overstates the evidence as it stands, in particular in the context of our revised colocalization analysis. We have amended the title accordingly.***

6. HLA-DRB1*15:01 has been well reported for leprosy susceptibility in several populations. What about this signal in your data? How about the LD between reported HLA 4-digit allele and it?

***Many thanks for this suggestion. Interestingly, DRB1*15:01 is very rare in our study samples, with no Malians carrying this allele, and only 2 Malawians (both cases). This has been added to the main text as follows (lines 196-201):***

“***HLA-DRB1*15:01 has been identified as a dominant determinant of leprosy risk in Chinese populations (Z. Wang et al., 2016), with confirmatory evidence of association in Vietnamese (Dallmann-Sauer et al., 2020) and Brazilian (Vanderborght et al., 2007) populations. In our study samples HLA-DRB1*15:01 carriage is rare, with only 2 individuals carrying HLA-DRB1*15:01 alleles in Malawi (both cases) and no HLA-DRB1*15:01 alleles observed in Mali.”***

Reviewer #2

Summary

Gilchrist et al performed the first leprosy GWAS in African populations and identified an African-specific leprosy-risk locus on chromosome 10q24.32. Moreover, five loci previously associated with leprosy in Asian populations were validated in Malians and Malawians. The GWAS analysis was well conducted. However, the interpretation of the findings requires further discussion.

***Many thanks for taking the time to review our work.***

Major Issues

1. In the Manhattan plot for the Malawi and Mali populations combined (Fig 1), it appears to be a suggestive locus on chromosome 3. However, I could not find SNPs with the same magnitude of meta-analysis p-value for chromosome 3 on Table S1. This needs to be clarified. If the signal is correct, what are the potential genes of interest in the chromosome 3 locus? The signal appears to be stronger than the HLA locus in Africans.

***Many thanks for highlighting this.*** ***Supplementary Table 1 describes suggestive results from the GWAS in Malawian samples, alongside evidence for replication at these loci in Mali. We agree that it is of interest to report suggestive associations in the meta-analysis (even if the evidence in Malawi alone is weak) and we have added an additional supplementary table (Table S2) to address this. This includes the chromosome 3 association at ZNF385D.***

2. Although stigmatized, leprosy is not a deadly disease as tuberculosis was during middle age. What would be the rationale to conclude that leprosy caused selective pressure for atopic and autoimmune diseases in modern populations as implied in the abstract and discussion? For example, if leprosy mortality was high, protective alleles (candidates for autoimmunity risk) could have been positive selected. Since mortality was likely the same as in non-leprosy cases one would expect neutrality. In that case, social and environmental factors may have played an important role. While this discussion falls beyond the current study, the authors need to extensively substantiate their discussion regarding leprosy as the cause of selection or tone down the claim.

***We agree that any selection pressure imposed by leprosy is unlikely to have operated through early mortality. It is possible that leprosy operated as an agent of selection through reduced fertility, particular among patients with lepromatous disease. We have expanded our discussion to reflect this (lines 302-310) and have also modified our language to make it clear that leprosy needn’t be the sole (or even major) selective agent at this locus.***

3. eQTLs can be cell-specific but they can also differ between populations and regarding their response to stimulus across populations (Nédélec, Y et al, Cell 2016 and Randolph, HE et al, Science 2021). The eQTL effect for ACTR1A in CD4+ T cells was detected in Estonians, while the leprosy GWAS signal was observed in Africans. Although I agree that ACTR1A can be a good candidate gene, one cannot disregard the potential role for other genes at the chr10 locus. This should be addressed as a limitation.

***Very many thanks for highlighting this important point. We having added the following to the discussion (lines 329-341):***

***“It is noteworthy, however, that there are limitations to our regulatory mapping in this context. Performing colocalisation between a leprosy GWAS in African-ancestry individuals and eQTL mapping data from individuals of European ancestry makes the assumption that the underlying causal regulatory determinants are shared between populations. While population-specific eQTLs are uncommon (Raj et al., 2014), they are well described (Nedelec et al., 2016). Similarly, the observation that the leprosy associated signal colocalises with two different genes in different tissues highlights the high levels of regulatory activity at this locus, and it remains plausible that leprosy susceptibility at this locus operates in a tissue or context not assessed here, and that pleiotropy with other traits at this locus could operate through distinct regulatory mechanisms.”***

4. Since the identification of ACTR1A as a leprosy candidate gene was based on eQTLs in Europeans, are the leading GWAS SNPs eQTLs for other genes in databases such as GTEx? What about the eQTL effect in other tissues or cell populations? Is ACTR1A differentially expressed in leprosy patients? Are the leading SNPs on chr10 response eQTLs for M. leprae (Manry, J et al, PLoS Gen 2017)? There are multiple publicly available datasets (i.e. GEO) that include gene expression profiles in leprosy affected cases and healthy controls (whole blood, different cell types or leprosy lesions). These sources can be evaluated to strengthen ACTR1A as the best candidate gene at the chr10 locus.

***Many thanks for these very helpful suggestions. We have extended our eQTL analysis to include colocalization analysis with eQTL in skin and peripheral nerves in GTEx. Interestingly this analysis suggests colocalization of the leprosy signal with eQTLs for TMEM180 in addition to ACTR1A in CD4+ T cells (lines 142-147, which we also illustrate in a revised Figure 3):***

***“In skin and peripheral nervous tissue, however, there is evidence for colocalisation between the leprosy risk locus and an eQTL for TMEM180 expression (Figure 3B, PP4 = 0.92 and PP4 = 0.95 respectively), with the leprosy protective allele being associated with reduced expression of TMEM180 in both cell types (skin – sun unexposed, p = 1.84 × 10−10, β = −0.174; tibial nerve, p = 1.07 × 10−59, β = −0.596).”***

***We now use transcriptomic datasets as suggested to test the candidacy of both ACTR1A and TMEM180 as potential mediators of leprosy risk (lines 149-179, and revised Figure 3):***

***“Having identified two distinct candidates in ACTR1A and TMEM180 for mediation of the leprosy risk effect at chromosome 10q24.32, we sought to explore the expression of both genes in the context of leprosy. To do this we downloaded publicly-available gene expression data in whole blood and skin from patients with leprosy and healthy controls (Belone et al., 2015; Manry et al., 2017; Montoya et al., 2019; Tio ́-Coma et al., 2021), exploring evidence for differential expression of ACTR1A and TMEM180 in leprosy. There is no evidence of differential gene expression of ACTR1A (p = 0.747) or TMEM180 (p = 0.069) in the whole blood of household contacts of leprosy in Bangladesh who do not develop disease (n=40) and those that do (n=40), either before onset of disease or at diagnosis (Fig. S4) (Tio ́-Coma et al., 2021). In keeping with this, genotype at rs2015583 is not associated (p < 0.05) with expression of any gene in cis in the whole blood of Vietnamese borderline leprosy patients (n=51) either unstimulated or following stimulation with sonicated M. leprae (Fig. S5) (Manry et al., 2017).***

***We then explored whether expression ACTR1A or TMEM180 was perturbed in leprosy skin lesions. In comparison to healthy skin samples (n=9), gene expression of ACTR1A is significantly down-regulated in skin lesions (n=67) from patients with leprosy in Brazil (Figure 3D, p = 0.0004) (Belone et al., 2015). In these data there is no evidence for differential expression of TMEM180 in leprosy skin lesions (Figure 3D, p = 0.822). To further explore the down-regulation of ACTR1A expression in leprosy skin lesions, we investigated the effect of subtype of clinical leprosy on ACTR1A expression in the same dataset (Belone et al., 2015). In that analysis (Figure 3E) the down-regulation of ACTR1A expression in leprosy skin lesions compared to healthy controls is seen in tuberculoid (p = 0.0085) and borderline forms of leprosy (p = 0.0003), but not in lepromatous disease (p = 0.218). We replicated this finding in a complementary RNA-Seq dataset of leprosy skin lesions from individuals with lepromatous leprosy (n=9) and tuberculoid disease (n=6) (Montoya et al., 2019). In these data we again see significant down-regulation of ACTR1A expression in lesions of leprosy patients with tuberculoid disease compared to lepromatous disease (Figure 3F, p = 0.0007).”***

***The sum of the chromatin state, eQTL data and transcriptomic data is highly suggestive that leprosy risk in African populations is mediated by ACTR1A (rather than TMEM180 or another gene), however we have modified our language throughout (in particular in the discussion) to better reflect uncertainty in the identification of the effector of leprosy risk at this locus.***

***These additional analyses are also described in the Methods sections; “Functional annotation and pleiotropy” (lines 595-641), “Differential gene expression analysis in leprosy” (lines 643-678).***

5. Does any of the high LD SNPs in chr10 locus colocalize with transcription factor (TF) binding sites or disrupt TF binding motifs? Can the authors go a step further and identify the link between the genotype and the eQTL effect?

***Thank you for this suggestion. We now include an analysis of chromatin state overlap and transcription factor binding site disruption (lines 115-128) as follows:***

***“To understand whether the identified leprosy-associated variation at chromosome 10q24.32 was likely to operate through regulation of gene expression we explored overlap between SNPs in the 99% credible set (n=32) and functional chromatin states in nine cell types using ENCODE annotations (Table S7) (Ernst et al., 2011). Overlap of credible set SNPs with regulatory chromatin states is most marked in K562 cells (a myeloid leukaemia cell line), with 11 of 32 credible set SNPs overlapping with chromatin states suggestive of regulatory function; 7 with strong enhancers, 3 with weak enhancers and one with an active promoter. One 99% credible set SNP, rs2274351:T, lies within an active promoter in all nine cell types (chr10:104,260,000-104,265,601), which is located at the 5’ end of two genes; ACTR1A and SUFU. Moreover, rs2274351 is located within the binding motifs of two transcription factors; CNOT3 and TRIM28.”***

***These additional analyses are also described in the Methods section; “Functional annotation and pleitropy” (lines 595-641.***

6. The Malawi population has the lowest overall allele frequency for leprosy-risk SNPs in chromosome 10 compared to other populations, including Africans from 1k genomes. Are there any known signs of selection for variants in this chromosomal region?

***Very many thanks for this suggestion. We now include an analysis of integrated haplotype scores, providing evidence for recent positive selection at the locus. We report this in the text (lines 102-112) as follows:***

***“The identification of a pleiotropic locus for leprosy, autoimmune and atopic disease risk suggests a hypothesis in which selection pressure imposed by leprosy has shaped the evolution of immune-mediated disease in modern populations. To further explore this we evaluated evidence for recent positive selection at the identified locus, examining integrated haplotype scores (iHS) in 1,000 Genome Project populations. In that analysis, we find evidence of positive selection (genome-wide rank p<0.05) at a genomic region (chr10:104270877-104280877), which overlaps with the leprosy-associated locus, in 16 of 25 1,000 Genome Project populations (Supplementary Table 4), with the strongest evidence of selection (iHS>2) in 4 African ancestry populations (ASW, LWK, GWD and MSL).”***

***These additional analyses are also described in the Methods section; “Signatures of positive selection” (lines 587-593).***

7. Personal communications should be avoided in the final version of the manuscript. The authors referred to a lack of association of chr10 in and leprosy in the Chinese population. What about these SNPs in the Vietnamese or Indian populations?

***We have removed reference to this as a personal communication, thanking the investigators in the acknowledgements and citing their work. We now provide additional analysis of the role of rs2015583 in leprosy risk in Indian-ancestry populations. Interestingly, while there is no evidence for association between genotype and leprosy per se, there is evidence suggestive of a protective effect for rs2015583:G on multibacillary disease. We detail this as follows (lines 76-85, Figure 2C):***

***“To explore the role of genotype at rs2015583 in leprosy risk in India, we used previously-published genotyping data from a leprosy case-control collection of Indian-ancestry individuals recruited in New Delhi (Wong, Gochhait, et al., 2010). In 448 individuals (209 cases and 239 controls), genotype at rs2015583 is not associated with risk of leprosy overall (p = 0.299, OR = 0.80 (95% CI 0.52 − 1.22)). However, in a multinomial regression model, in which cases are stratified by disease subtype, genotype at rs2015583 is associated with risk of multibacillary disease (Figure 2C, p = 0.044, OR = 0.56 (95% CI 0.32 − 0.98)) but not paucibacillary disease (p = 0.692, OR = 1.11 (95% CI 0.66 − 1.86)).”***

***Given that the model of association (an effect restricted to a clinical subtype) here differs to what we observe in Africa, this needs to be interpreted with caution and we have added the following to the discussion (lines 266-273):***

***“Interestingly, while we observe no evidence of association with leprosy overall in Indian-ancestry individuals, we do see an effect of rs2015583 genotype on risk of multibacillary leprosy in this population, with an effect size and direction of effect consistent with that observe in African populations. This observation differs from our findings in African populations, where the effect of rs2015583 is not restricted to multibacillary disease, and the effect of this locus on leprosy risk in India will need to be clarified in future studies.”***

***These additional analyses are also described in the Methods section; “Association analysis at rs2015583 in non-African populations” (lines 543-573).***

Reviewer #3

Summary

The paper presents a GWAS analysis in Malawi and Mali screening leprosy patients and controls. Among the genes initially pinpointed, data focus on a novel candidate that is validated using public databanks for gene expression suggesting the association of rs2015583 of ACTR1 as an eQTL and also recovering datasets from case-controls in other diseases such as IBD, Atopy, and an interesting effect where the same SNP was associated, although in IBD in an opposite direction. Later, the authors replicated findings of previously associated genes and SNPs in these African populations. The paper is well written, and the authors have a long contribution to the genetics of infectious diseases with seminal published papers.

***Very many thanks for taking the time to review our work.***

Major Issues

The group has published data on tuberculosis GWAS studies in Africa and it would be helpful if the authors test ACTR1 also in these populations to evaluate whether the ACTR1 is a major gene involved in mycobacterial infections consequently the dynein-dynactin motor function of the cell.

***Many thanks for this suggestion. We now include an analysis exploring association of rs2015583 with tuberculosis in Ghana and The Gambia. We report this in the results as follows (lines 57-63):***

***“To understand whether genotype at rs2015583 could act as a determinant of mycobacterial disease in Africa more broadly, we examined the role of rs2015583 in genetic association studies of tuberculosis in Ghana (1,359 cases, 1,952 controls) and The Gambia (1,316 cases, 1,382 controls). In that analysis, genotype at rs2015583 is not associated with tuberculosis in Ghana (p = 0.746, OR = 1.02 (95% CI 0.92 − 1.03)) or The Gambia (p = 0.112, OR = 0.91 (95% CI 0.81 − 1.02)).”***

***These additional analyses are also described in the Methods section; “Association analysis at rs2015583 in tuberculosis” (lines 575-585).***

The Malian and Malawian case-control populations have been studied before. It is important to clarify whether all samples from previous studies were used in this work. The cases numbers are very different from previous publications at least as described in Fitness (2004) and Meisner (2001). So, retrieving information from these previous publications does not help in the understanding distribution of the cases according to some key variables according to clinical form outcome. Thus, the distribution of cases between clinical forms (PB and MB) along with age at diagnosis and gender should be included. African populations generally have a higher proportion of PB patients. Although heterogeneity analysis did not show any differences from the clinical forms, this information would be helpful for readers. Also, information on reactional states (% of ENL and RR) is important. Maybe a supplementary table could be introduced.

***Very many thanks for highlighting that this needed more clarity. We have included a new Table 1 in main manuscript to provide these data.***

Also, in 2010, the same group published a paper (Wong et al NEJM) replicating the findings of the Chinese GWAS and found LACC (formerly C13orf31, rs3764147, Ile254Val) in Mali. So, in this case, it seems that the data is already published. This part should be clarified. A possibility should at the metanalysis declare that this data is already published if this is the case.

***Many thanks. We have indeed used the Mali samples to replicate the leprosy association at LACC1, we now make this clear in the results reported the replication of non-HLA leprosy associations (lines 210-212):***

***“We have previously published a replication analysis at rs3764147 (the leprosy-associated SNP at LACC1) in the Mali samples”***

In this regard, the same paper (Wong et al, 2010) had genotyped 492 patients in New Delhi and 382 in Kolkata for GWAS replication. It should be very interesting to have validation of the findings of this paper (ACTR1) using these Indian populations.

***Very many thanks for this suggestion. We now include a replication analysis at rs2015583 using the New Delhi samples. We detail the results of this in the manuscript as follows (lines 76-85):***

***“To explore the role of genotype at rs2015583 in leprosy risk in India, we used previously-published genotyping data from a leprosy case-control collection of Indian-ancestry individuals recruited in New Delhi (Wong, Gochhait, et al., 2010). In 448 individuals (209 cases and 239 controls), genotype at rs2015583 is not associated with risk of leprosy overall (p = 0.299, OR = 0.80 (95% CI 0.52 − 1.22)). However, in a multinomial regression model, in which cases are stratified by disease subtype, genotype at rs2015583 is associated with risk of multibacillary disease (Figure 2C, p = 0.044, OR = 0.56 (95% CI 0.32 − 0.98)) but not paucibacillary disease (p = 0.692, OR = 1.11 (95% CI 0.66 − 1.86)).”***

***These additional analyses are also described in the Methods section; “Association analysis at rs2015583 in non-African populations” (lines 543-573).***

The validation of ACTR1 and functional analysis could be improved if the authors retrieve from public datasets to better understand how M. leprae regulates ACTR1 expression (and maybe the other gene in the locus) irrespective of the SNP. First, RNASeq and microarray datasets are available in GEO or Array express mostly testing skin biopsies (Montoya et al 2009, Belone et al 2015, Lee et al 2010, among others). In addition, Schwann cells or macrophages infected with live M.leprae have been tested (Masaki et al 2013, Toledo-Pinto 2016, Leal-Calvo et 2021a). Novel studies are also available using single cells RNASeq ou spatial mapping.

Another possibility would be to use whole blood, PBMCs, or skin biopsies from leprosy patients (preferably genotyped) to evaluate gene expression and eQTL of ACTR1 in leprosy.

Also, a dataset for eQTL presenting data from whole-blood cells challenged with sonicated M. leprae (Manry et al, PLoS Genetics, GSE100853) is publicly available and could confirm the impact of this SNP on ACTR1 expression in the leprosy context. Also, other SNPs in the genes nearby could be used.

***Many thanks for these suggestions, which are very much in keeping with Reviewer #2’s suggestions above. We have extended our eQTL analysis to include colocalization analysis with eQTL in skin and peripheral nerves. Interestingly this analysis suggests colocalization of the leprosy signal with eQTLs for TMEM180 rather than ACTR1A (lines 142-147, which we also illustrate in a revised Figure 3):***

***“In skin and peripheral nervous tissue, however, there is evidence for colocalisation between the leprosy risk locus and an eQTL for TMEM180 expression (Figure 3B, PP4 = 0.92 and PP4 = 0.95 respectively), with the leprosy protective allele being associated with reduced expression of TMEM180 in both cell types (skin – sun unexposed, p = 1.84 × 10−10, β = −0.174; tibial nerve, p = 1.07 × 10−59, β = −0.596).”***

***We further used the Manry et al. dataset to test for leprosy-specific eQTLs at the chromosome 10 locus in whole blood (lines 159-162):***

***“genotype at rs2015583 is not associated (p < 0.05) with expression of any gene in cis in the whole blood of Vietnamese borderline leprosy patients (n=51) either unstimulated or following stimulation with sonicated M. leprae (Fig. S5) (Manry et al., 2017).”***

***We now use transcriptomic datasets as suggested (Montoya et al, Belone et al, Tio-Coma et al.) to test the candidacy of both ACTR1A and TMEM180 as potential mediators of leprosy risk (lines 163-179, and revised Figure 3):***

***“We then explored whether expression ACTR1A or TMEM180 was perturbed in leprosy skin lesions. In comparison to healthy skin samples (n=9), gene expression of ACTR1A is significantly down-regulated in skin lesions (n=67) from patients with leprosy in Brazil (Figure 3D, p = 0.0004) (Belone et al., 2015). In these data there is no evidence for differential expression of TMEM180 in leprosy skin lesions (Figure 3D, p = 0.822). To further explore the down-regulation of ACTR1A expression in leprosy skin lesions, we investigated the effect of subtype of clinical leprosy on ACTR1A expression in the same dataset (Belone et al., 2015). In that analysis (Figure 3E) the down-regulation of ACTR1A expression in leprosy skin lesions compared to healthy controls is seen in tuberculoid (p = 0.0085) and borderline forms of leprosy (p = 0.0003), but not in lepromatous disease (p = 0.218). We replicated this finding in a complementary RNA-Seq dataset of leprosy skin lesions from individuals with lepromatous leprosy (n=9) and tuberculoid disease (n=6) (Montoya et al., 2019). In these data we again see significant down-regulation of ACTR1A expression in lesions of leprosy patients with tuberculoid disease compared to lepromatous disease (Figure 3F, p = 0.0007).”***

***The sum of the chromatin state, eQTL data and transcriptomic data is highly suggestive that leprosy risk in African populations is mediated by ACTR1A (rather than TMEM180 or another gene), however we have modified our language throughout (in particular in the discussion) to better reflect uncertainty in the identification of the effector of leprosy risk at this locus.***

***These additional analyses are also described in the Methods sections; “Functional annotation and pleiotropy” (lines 595-641), “Differential gene expression analysis in leprosy” (lines 643-678).***

Minor Issues

The replication strategy of former associated genes and SNPs should be clarified. How SNPs/genes were selected? The approach to defining the 34 SNPs should be described in the methods. There are genes and SNPs identified in Chinese GWAS, exome, and other studies, but why other GWAS were not considered? Some of the SNPs tested are non-informative with frequencies of 0-0.1% in Africans, such as IL23, CCDC88B, FLG, CARD9, CCDC88B, GIT2, ALDH2, HIF1A, IL12B, TCN2 (Supp Table 6), and they should be excluded. A rapid and previous analysis evaluating the frequencies in Africans should be included as the first step of validation. Data on TLR1 from the same group and relevant genes such as PRKN, LRRK2, other SNPs from NOD2 (702W- rs2066844, rs5743291).

***Many thanks for highlighting that this was not sufficiently clear.*** ***We now clarify the gene/SNP selection strategy, and have added the suggested loci falling outside our original approach. We now cite 10 genetic association studies in which each assessed variant was first reported (see text and Table S10), and have added the following to the text (lines 205-210):***

***“We thus sought to replicate evidence for leprosy association at genetic loci outside the HLA associated with leprosy risk in other populations. In this analysis we included variants associated with leprosy susceptibility at genome-wide significance in other populations, alongside variants at LRRK2 and PRKN, and pathogenic variants at NOD2.”***

***We have also adopted the suggested step-wise approach to tested for association at these loci, adding the following to the text (lines 212-218):***

***“Of 37 leprosy risk loci assessed, 3 were not reliably imputed in our dataset (rs145562243, rs6871626, rs1873613) and 10 (rs146466242, rs925368, rs671, rs149308743, rs142179458, rs75680863, rs2066844, rs76418789, rs5743291, rs663743) were monomorphic or at very low frequency (MAF<1%) in our study samples (Supplementary Table 6). We were thus able to assess replication at 24 loci, at which our study had adequate power (>80%) to replicate (p<0.05) findings at 7 loci.”***

In addition, since rs2015583 is associated with leprosy and IBD in opposite directions, gene expression in other datasets could bring clarity to how ACTR1 levels would function in these diseases. Curiously, most of the genes previously associated with both diseases indicate association in the same direction, especially for NOD2 SNPs.

***Very many thanks. As above, we have now extended our analysis of eQTL mapping datasets to include skin and peripheral nerve tissue from the GTEx project. This analysis suggests colocalization of the leprosy signal with eQTLs for TMEM180 rather than ACTR1A (lines 142-147, which we also illustrate in a revised Figure 3):***

***“In skin and peripheral nervous tissue, however, there is evidence for colocalisation between the leprosy risk locus and an eQTL for TMEM180 expression (Figure 3B, PP4 = 0.92 and PP4 = 0.95 respectively), with the leprosy protective allele being associated with reduced expression of TMEM180 in both cell types (skin – sun unexposed, p = 1.84 × 10−10, β = −0.174; tibial nerve, p = 1.07 × 10−59, β = −0.596).”***

***In addition, we now extend our discussion to acknowledge that, in a region of dense regulatory activity, pleiotropy at this locus could have distinct, tissue-specific regulatory consequences in different diseases/traits (lines 335-341).***

In supplementary Figure 2, there are some SNPs passing the threshold in Malawian or Malian populations, although probably did not reach significance when the populations were combined. Nevertheless, it should be mentioned at least the legends of the supplementary Figure.

***Very many thanks. We have added a new Table S2 which lists any variant with suggestive significance in the meta-analysis. We have also added the numbers of variants exceeding suggestive and genome-wide significance in each population in the legend of Figure S2.***

Genomic structure in the PCA (sup Fig 8) also indicates an interesting pattern where populations are clustered, which means that some SNPs can indeed be important in Malawian but not Malian or vice-versa. In this context, it is curious that no signal at 10q24 was detected in these populations separately.

***Many thanks for this. We agree that there is evidence of considerable population structure between the Malawi and Mali samples. We don’t have sufficient power to fine map the association at 10q24 in each population separately. Nonetheless, for every SNP associated with leprosy p<10^-5^ in Malawi (n=18), we see evidence of replication in Mali, with remarkably consistent effect estimates (Table S1). With our current sample size we are not able exclude independent signals within each population, however given the stable effects across the two populations it seems very likely that there is a common single causal variant here. Given how distinct the two populations are, performing meta-analysis across these two populations, especially in a fixed-effects model, may have compromised study power for variant discovery at 10q24 and other loci. We acknowledge this limitation in the discussion (lines 286-290) as follows:***

***“It is also important to note that Malawi and Mali are distinct populations, and fixed-effects meta-analysis without modelling inter-population ancestry differences, for instance with MR-MEGA, may have compromised study power.”***
